# 

**Published:** 1903-05-16

**Authors:** 


### NOTICE TO CORRESPONDENTS.

All MSS., Letters, Books for Review, and other matters intended for the Editor should be addressed The Editor, "The Hospital," The Hospital Buildings, 28 & 29 Southampton Street, Strand, W.C.

The Editor cannot undertake to return rejected MS., even when accompanied by stamped directed envelope.

### Notice to Advertisers and Subscribers.

All Advertisements, Orders for Copies of The Hospital, and Business Communications should be addressed to **THE MANAGER, (Not to the Editor)**, The Hospital, 28 & 29 Southampton Street, Strand, London, W.C.

The Cost of Subscription, post free, is as follows.—Per week, 3½d.; per quarter, 3s. 9d.; per half-year, 7s. 6d.; per annum, 15s. Foreign and Colonial:—Per annum, 19s. 6d., payable, in all cases, in advance.

**NOTICE.—Vol. XXXIII. of "The Hospital" (October 1902 to March 1903) in handsome cloth binding, will shortly be on sale at the office of this Journal, price 7s. 6d. Cases for binding the Numbers contained in this Volume 1s. 6d. Index to Vol. XXXIII., 2½d., post free.**

**The monthly part for May is now ready. Price 1s., by post 1s. 3d.**

### BOOKS RECEIVED.

Health Resorts Bureau.

"Carlsbad and its Therapeutical Importance."

A. and C. Black.

"Sour Music." By J. N. Carlyle.

Baillière, Tindall and Cox.

"The Imperfectly Descended Testes." By W. McAdam Eccles.

Sampson Low and Co.

"A Complete System of Nursing." By Honnor Morten.

R. L. Sharland.

"The Scientific Roll." "Bacteria," Vol. I., No. 7.

### Scraps and Gleanings.

Birds' nests have been found in a dust-bin, a tool-bag, and an old hair-broom in a garden at Bourne End.

The Scottish Patriotic Association has made a profit of £14 odd on the sale of medals issued as a protest against the title "Edward VII."

The Bute marriage dowry—being the interest on a sum of £1,000 invested by the late Marquis of Bute for the purpose of providing a sum of money annually to enable a poor deserving girl to furnish a home—has this year been awarded to the newly-made wife of a coachbuilder's assistant. It amounted to £30 14s.

The Grand Duchess of Saxe-Coburg and Gotha will lay the foundation stone of the Victoria Memorial Hospital at Nice on May 7th.

### The Student of Medicine.

### Appointments.

**Richard Ahern**, M.B., Ch.B.R.U.I., appointed Resident Medical Officer to the Tooting Home Infirmary of the Wandsworth and Clapham Union. **W. T. D. Allen**, M.B., B.Ch., B.A.O.R.U.I., appointed Honorary Assistant Surgeon to St. George's Hospital for Diseases of the Skin, Liverpool. **William Turnbull Barrie**, M.B., C.M.Edin., appointed Certifying Factory Surgeon for the Hawick District of the county of Roxburgh. **John M. Bowie**, M.D., M.R.C.P.Edin., appointed District Medical Officer of the Edinburgh Parish Council. **H. Case**, L.R.C.P., L.R.C.S.Edin., appointed District Medical Officer of the Chorley Union. **A. H. Clarke**, M.R.C.S.Eng., appointed Honorary Pathologist to the General Hospital, Hobart, Tasmania. **William A. Conlon**, M.B.Syd., appointed Public Vaccinator for the District of Reefton, New Zealand. **Conrad C. J. Erhardt**, M.R.C.S., L.R.C.P.Lond., appointed Certifying Factory Surgeon for the Crosshills District of the county of Yorks. **G. A. Fischer**, M.B., B.S.Adel., appointed Physician in Charge of the Nose and Throat Department of the Adelaide Hospital. **A. Knyvett Gordon**, M.B., B.C., B.A.Cantab., appointed Lecturer on Infectious Diseases at Owens College (Victoria University), Manchester. **F. Clayton Gould**, L.R.C.P., L.R.C.S.Edin., L.F.P.S.Glasg., appointed Medical Examiner to the Pioneer Life Assurance Company, Liverpool. **M. H. Grattan**, L.R.C.P., L.R.C.S. Irel., appointed Certifying Factory Surgeon for the Ongar District of the county of Essex. **William Ernest Hills**, M.R.C.S.Eng., L.R.C.P.Lond., appointed Medical Officer to the Hampstead Division of the London Postal Service. **F. I. M. Jupe**, B.A.Camb., L.S.A., appointed Certifying Factory Surgeon for the Histon District of the county of Cambridge. **N. Y. Lower**, M.R.C.S., L.R.C.P.Lond., appointed Certifying Factory Surgeon for the Presteign District of the county of Radnor. **Hugh A. McCleland**, M.R.C.S.Eng., appointed Port Health Officer for the Port of New Plymouth, New Zealand. **W. Murray**, M.D.Edin., appointed Certifying Factory Surgeon for the Hessle District of the county of Yorks. **A. J. W. Philpott**, M.B., Ch.B.Melb., appointed Senior Medical Officer of the Yarra Bend Asylum. **R. R. Sutter**, M.D., M.S.Aberd., appointed Certifying Factory Surgeon for the Warboys District of the county of Hunts. **A. Landon Whitehouse**, M.R.C.S., L.R.C.P., L.D.S.Eng., appointed Dental Surgeon to the Westminster General Dispensary. **C. S. Willis**, M.B., M.Ch.Syd., appointed Health Officer for Lennonville, West Australia.

### "The Hospital" Institutional Library.

"Hospitals and Asylums of the World." 4 Vols., with a Portfolio of Plans, complete £12 12s.

"Burdett's Hospitals and Charities, 1902." 5s.

"Burdett's Official Nursing Directory, 1903." 3s. net.

"Cottage Hospitals, General, Fever, and Convalescent." 10s. 6d.

"Uniform System of Accounts for Hospitals and Public Institutions." New Edition. 4s. net.

"Hospital Expenditure: The Commissariat." 2s. 6d.

"A Legal Handbook for the Use of Hospital Authorities." 2s. 6d.

"The Cottage Hospital Case Book or Register of Patients." 10s. and 12s. 6d.

"The Furnishing and Appliances of a Cottage." 6d.

All these are published by the Scientific Press, Ltd., and may be obtained through any bookseller, or direct from the publishers, 28 and 29 Southampton Street, Strand, London, W.C.

### The Untrained

***versus***

### The Trained Nurse.

The untrained nurse is no longer countenanced, we are rapidly leaving the days of Mrs. Gamp far behind us. It is necessary therefore that the nurse aspirant should before entering upon this important calling obtain the fullest particulars as to **how and where to train** in order to become an efficient nurse. In

### THE NURSING PROFESSION,

Price **2/4** post free,

By Sir HENRY BURDETT, K.C.B.,

The would-be nurse will find **all** the information she needs in deciding upon the Institution at which to go through her training and to find employment afterwards. The book is up to date in all respects, and is in fact indispensable to anyone wishing to become a nurse and to be properly trained.

### Aids to Training.

### NURSING: ITS THEORY AND PRACTICE,

By Percy G. Lewis, M.D., price **3/6** post free,
Is a complete Text-book of Medical, Surgical and Monthly Nursing.

### ELEMENTARY ANATOMY AND SURGERY FOR NURSES,

By William McAdam Eccles, M.S. Lond., M.B., price **2/6** post free,
Is invaluable to Nurses who are beginning the study of Anatomy.

### ELEMENTARY PHYSIOLOGY FOR NURSES,

By C. F. Marshall, M.D., B.Sc, F.R.C.S., price **2/-** post free,
Contains all that Nurses require to know of this subject.

### THE NURSE'S DICTIONARY OF MEDICAL TERMS,

By Honnor Morton, price **2/-** post free,
Should always be carried in the apron pocket for ready reference.

*These books are obtainable of all Booksellers and are published by*
THE SCIENTIFIC PRESS, LIMITED, 28 and 29 Southampton Street, Strand, London,
*who will send their latest Catalogue of Nursing Text-books post free to any Nurse on application.*

